# Continuous evolution of HIV-1 more than ten years after infection in an elite neutralizer

**DOI:** 10.1186/1742-4690-9-S2-P45

**Published:** 2012-09-13

**Authors:** A Chaillon, M Braibant, S Hué, S Bencharif, A Moreau, D Enard, A Samri, H Agut, F Barin

**Affiliations:** 1 INSERM U966 Resaerch Unit, F. Rabelais University, Tours, France; 2 INSERM U966 Research Unit, F. Rabelais University, Tours, France; 3 Centre for Medical Molecular Virology, University College London, London, UK; 4 Ecology and Evolution Laboratory, CNRS UMR 7625, ENS, Paris, France; 5 INSERM U945, Pierre et Marie Curie University, Paris, France; 6 Virology Laboratory, Pitié-Salpétrière Hospital, Paris, France

## Background

The viral evolution of HIV-1 and its escape to autologous neutralizing antibodies (Nabs) during the early years of infection have been analyzed in depth. In contrast, little is known about neither the long-term evolution of the virus in patients who developed broadly Nabs (bNabs) nor the mechanism of escape in presence of these bNabs.

## Methods

We have studied the viral population infecting an HIV-1 infected long term non progressor (LTNP) who had developed Nabs toward all tier 2/3 viruses (6 clades) tested, 9 years after infection, and was then followed up over 7 years. Sixty-nine env clones issued from sequential blood samples collected from 9 years to 16 years post-infection were obtained. Thirteen infectious clones representative of the genetic diversity of variants present at the different time-points were selected. Pseudotyped viruses harboring these different envelopes were generated and their sensitivity to neutralization was analyzed.

## Results

Evidence of ongoing viral evolution was found, supported by both the phylogenetic analyses that showed a continuous diversification and an increasing divergence overtime. The mean autologous neutralization titers of the sequential sera toward the 13 env variants significantly increased during the period of late follow-up. The env pseudoviruses displayed a broad range of sensitivity to the autologous sera, with the most resistant variant identified at the last visit suggesting that it represented a late emerging escape variant. We identified 5 amino acids substitutions that appeared associated with escape to bNabs. They were V319I/S, R/K355T, R/W429G, Q460E and G/T463E, in V3, C3 and V5 regions.

## Conclusion

This study showed that HIV-1 may continue to evolve in presence of both broadly neutralizing antibodies and increasing autologous neutralizing activity more than 10 years post-infection. Such material may provide opportunities to reveal the molecular determinants of escape of HIV-1 to highly potent broadly neutralizing antibodies.

